# Our New Pub. House

**Published:** 1889-01

**Authors:** 


### EDITORIALS AND MISCELLANEOUS.

OUR NEW PUBLISHING HOUSE.—The RECORD is now published at a new establishment—the job office of T. S. Reynolds, No. 24 S. Broad St.

Hereafter it will be issued more regularly and mailed on the first day of each month.

No one unacquainted with the business of Journalism has any conception of the difficulties often encountered in getting out a publication promptly. Our prospect in this particular, we feel safe in saying, is now better than we have ever had before, and with new type and new and energetic men at the helm, our Journal, though neat and creditable in the past, will be more so in the future.

